# First Detection of *Lactococcus petauri* in Domestic Dogs in Italy

**DOI:** 10.3390/ani14223279

**Published:** 2024-11-14

**Authors:** Simona Sciuto, Giuseppe Esposito, Paolo Pastorino, Khalid Shahin, Katia Varello, Eliana Trabunella, Giulia Milanese, Sonia Scala, Marino Prearo, Pier Luigi Acutis, Angelo Salerno, Simona Zoppi, Silvia Colussi

**Affiliations:** 1 Istituto Zooprofilattico Sperimentale del Piemonte, Liguria e Valle d’Aosta, 10154 Turin, Italy; simona.sciuto@izsplv.it (S.S.); paolo.pastorino@izsplv.it (P.P.); katia.varello@izsplv.it (K.V.); eliana.trabunella@izsplv.it (E.T.); giulia.milanese@izsplv.it (G.M.); sonia.scala@izsplv.it (S.S.); marino.prearo@izsplv.it (M.P.); pierluigi.acutis@izsplv.it (P.L.A.); simona.zoppi@izsplv.it (S.Z.); 2 PHARMAQ Analytiq UK Ltd., 8b Solasta House, Inverness Campus, Scotland IV2 5NA, UK; khalid.shahin@zoetis.com; 3 S.S. Microbiologia, Ospedale SS Antonio e Margherita, 15057 Tortona, Italy; asalerno@aslal.it

**Keywords:** animal health, antibiotic resistance, bacterial infections, canine infections, emerging pathogens, molecular diagnostics, pathogenesis, veterinary microbiology

## Abstract

*Lactococcus garvieae* has been considered for a long time the only causal agent of lactococcosis. In recent years, different papers reported the involvement of other two bacterial species: *Lactococcus petauri* and *Lactococcus formosensis*. A different host tropism has been described for these species where *L. garvieae* and *L. petauri* are predominant species in fish and humans’ infections, while *L. formosensis* in bovine. *L. garvieae* has been reported as rare infectious agent in dog. This paper represents the first isolation of *L. petauri* in two domesticated dog cases from urine and skin samples, respectively. The recovered *L. petauri* has been identified using PCR and sequencing based on Internal Transcribe Spacer (ITS) and phylogenetic analysis showed that it belongs to the *L. petauri* cluster with a 100% of identity with sequences previously reported from fish isolates while there were differences with *L. petauri* isolated from urinary tract infection from humans. *L. petauri* in human infection has been considered not necessarily deriving from the ingestion of contaminated food but rather as an opportunistic pathogen colonization intestinal tract. Differences among virulotypes have been reported for humans and dogs, and a comparison was also made between the virulotyping of *L. petauri* and *L. garvieae* in dogs. The antimicrobial pattern showed susceptibility for the election treatment molecules. These data contribute to our understanding of the host trophism of this species which was misclassified for long time and provide new data on its virulence factors and antimicrobial resistance.

## Simple Summary

*Lactococcus petauri* has been described for the first time in 2017 in a sugar glider. A few years later, it has emerged as important pathogen for fish and more recently it has been reported also in terrestrial mammals and humans. A lot of these cases were misdiagnosed as *Lactococcus garvieae* infection due to the limitation of the available common standard diagnostic techniques used which were unable to discriminate between these two species and relatively high similarity of the two pathogens at genetic and phenotypic levels. Till today, there are only two reported cases of lactococcosis in dogs with no previous cases were reported from Italy. This report is the first description of *L. petauri* infection in two domestics dog cases from Italy. The results of the current report provide an update on the host tropism, and additional knowledge on virulence factors and antimicrobial resistance of *L. petauri*.

## 1. Introduction

Lactococcosis is one of the most important bacterial diseases impacting aquaculture productions worldwide. This disease causes substantial economic losses for the fish farming sector due to high mortality rates and indirect costs related to treatment and prevention [1]. Historically *Lactococcus garvieae* has been regarded as the only causal agent of lactococcosis. However, recent studies have identified two additional bacterial species involved in the disease, *Lactococcus petauri* and *Lactococcus formosensis,* whose role was previously overlooked due to misidentification using common standard diagnostic techniques [2]. Recent evidence suggests that isolates previously classified as *L. garvieae* serotype II should now be attributed to *L. formosensis*, a pathogen which was firstly identified in fermented broccoli in 2014 [3,4]. On the other hand, *L. petauri* was isolated from sugar glider abscess in 2017 [5]. Numerous retrospective studies conducted after the discovery of new species within the *Lactococcus* genus re-assigned *L. garvieae* species to the species *L*. *petauri* and *L*. *formosensis* [6,7].

Most of available data on distribution, pathogenicity and host tropism are related to *L. garvieae*. This pathogen is known for its virulence and its ability to infect a wide range of freshwater and marine fish species [8]. Outbreaks are often triggered by abiotic factor such as water temperature typically above 15 °C. Infected fish exhibit external and internal hemorrhages exophthalmia, hyper melanosis and lethargy. The treatment of the disease is often ineffective due to the issue of multi-drug resistance associated with this pathogen [9].

While *L. garvieae* was known for the last decade as a significant fish pathogen, it has been reported to infect reptiles and mammals, including humans. In mammals, *L. garvieae* has been reported in cow’s mastitis cases and from infected milk, in swine’s blood, and in various meat products [1]. In rare cases, it was reported in wild bottlenose dolphins [10], in dogs, associated with tonsil colonization and respiratory issues [11,12], cats, horses, camel, turtle, snake, mugger crocodile [13] and in a central bearded dragon [14]. In humans, it has been described as opportunistic pathogen of immunocompromised or elderly patients often associated with co-morbidity factors [15] but recurrent penicillin resistant tonsillitis in children have also been recently described [16]. In humans, a wide range of symptoms have been reported such as septicemia [17], endocarditis [18] and urinary tracts infections [19]. A different host trophism has been described for *L. garvieae* and *L. petauri*, for which the predominant hosts were found to be fish and humans, while bovines are the predominant hosts for *L. formosensis*. Moreover, different virulence characters were reported in association with these three bacterial species [7].

Due to the improvement of diagnostic tools, the number of human infections report increased in recent years redefining the role of *L. garvieae* as potential zoonotic agent related to the consumption of raw fish, milk and dairy products [20]. Methods based on 16S rRNA gene sequencing and biochemical identification, including API20, BIOLOG, matrix-assisted laser desorption ionization-time-of-flight mass spectrometry (MALDI-TOF MS) have widely been applied for bacterial identification. However, these tools showed insufficient discriminatory power to distinguish among the three reported species due to the high phenotypic and genetic similarity [2,21,22].

With the evolution of advanced molecular and genomic tools, new data have been reported for *L. petauri* and *L. formosensis* using novel molecular diagnostic techniques such as Whole genome sequencing (WGS) [2,21,23], *ITS* sequencing [22], *gyr B* sequencing [24], multiplex PCR [25,26] and Multi Locus Sequence Analysis (MLSA) [27]. These new tools provided the opportunity to re-classify isolates previously identified as *L. garvieae* to *L. petauri* and *L. formosensis* from trout, human bacteremia, stool and fecal microbiota, meat sources, fermented milk, cheese, and dairy products [7]. Using these diagnostic tools also new isolates were correctly identified and this led us to describe the first case of human urinary tract infection sustained by *L. petauri* in 2023 using *ITS* sequencing [28].

To date, only two cases of *Lactococcus* infections in dogs are available, and both reported that the infections were caused by *L. garvieae* [11,12]. Pot et al. [11] described the phenotypic identification of *Lactococcus* strains from various animals using classical biochemical methods and SDS-PAGE, isolating *L. lactis* and *L. garvieae* from dog tonsils. In contrast, Thiry et al. [12] focused on a single case of a 16-year-old mixed breed dog with chronic upper and lower respiratory issues. The identification of the strain in the last case was based on MALDI-TOF MS and whole genome sequencing. Additionally, putative virulence genes of this strain were compared with those of *L. garvieae* isolated from different sources.

The aim of this study was to describe and characterize *L. petauri* isolated in two domesticated dogs from urine and skin samples, respectively. Moreover, a comparison between human and canine isolates derived from urine was carried out. Additionally, a data mining analysis based on PubMed records was conducted to explore the evolution of research on lactococcosis over time, identifying key areas of interest and emerging trends.

## 2. Materials and Methods

### 2.1. PubMed Database Analysis of Lactococcus spp.

The PubMed database was utilized, encompassing over 37 million citations from biomedical literature sourced from MEDLINE, scientific journals, and online books. Advanced search techniques were employed using the PubMed Advanced Search Builder with the keyword “*Lactococcus garvieae*” OR “*Lactococcus petauri*”. These specific keywords were chosen to avoid narrowing down the number of relevant studies and to focus on species of primary interest in aquaculture, such as *L. garvieae*, *L. petauri*, and *Lactococcus formosensis* [6,7,15,29]. This approach highlighted an increase in infection cases caused by these species, affecting both aquatic species like trout and terrestrial animals and humans.

For *L. garvieae*, the search spanned from 1991 to 2024, resulting in a total of n = 486 documents initially identified. After filtering for English language publications, the dataset was refined to n = 483 documents (all article type), encompassing studies involving both humans and other animals. Similarly, the search for *L. petauri* covered the period from 2017 to 2024, identifying a total of 17 documents. The datasets for both species were saved in PubMed format (.txt).

Subsequently, the saved files were uploaded to VOSviewer (version 1.6.20) for analysis [30]. Co-occurrence analysis was chosen to determine the correlation between items based on the number of documents in which they appear together. To ensure a more standardized view less subject to terminological variability, facilitating data aggregation and comparison, “MeSH keywords” was selected as the unit of analysis. The minimum number of occurrences for a keyword was set to 2 or 1.

### 2.2. Case Descriptions

Anamnestic data for two domestic dogs from Novara (Italy), were collected. The first dog is a 10-year-old female Newfoundland and the second one is a 6-year-old male mixed breed. The clinical history of both cases revealed clinical signs of supraventricular tachycardia and abdominal swelling in case 1 and interdigital nodules in case 2.

### 2.3. Microbial Identification

Urine sample from case 1 (ID #49222) and skin tissue sample from case 2 (ID # 50963) were aseptically collected and processed for standard aerobic, anaerobic, and microaerobic (5% CO_2_) bacteriology. The samples were then plated onto Columbia Blood Agar (CBA) (Liofilchem, Roseto degli Abruzzi (TE), Italy), McConkey Agar (MAC), and Chocolate Agar (CHOC) plates. Plates were incubated at 37 ± 1 °C and checked daily for 2 and 5 days for aerobic/microaerobic and anaerobic, respectively. Suspected colonies were subcultured on CBA plates at same conditions mentioned above followed by identification by VITEK^®^ MS (BioMérieux SA, Marcy l’Etoile, France), and MALDI-TOF MS (Bruker Daltonics, Bremen, Germany).

### 2.4. Antibiotic Susceptibility

Antibiotic susceptibility of the recovered bacteria was tested using the minimum inhibitory concentrations (MICs) method according to the guidelines of the Clinical and Laboratory Standards Institute (CLSI) [31,32]. Quality controls of the plates used for MIC were performed according to Table 5 of the CLSI VET01S supplement [33]. MIC breakpoints (expressed in mg/mL) were evaluated, and interpretative criteria were retrieved from Table 12 of the CLSI M45-ED3:2016 *Methods for Antimicrobial Dilution and Disk Susceptibility Testing of Infrequently Isolated or Fastidious Bacteria, 3rd Edition* [34]. For comparison, an isolate of *Lactococcus petauri* recovered from human urinary tract infection strain (33932 1LP_H, [28]), was included in the analysis.

### 2.5. DNA Extraction, Polymerase Chain Reaction and Sequencing of ITS 16S-23S Region

DNA was extracted using the boiling and freeze–thaw method. End-point PCR targeting the 16S-23S rRNA ITS region was carried out using the primers 16S 5′-GCTGGATCACCTCCTTTCT-3′ and 23S 5′-GGTACTTAGATGTTTCAGTTCC-3′ reported by [35]. PCR was performed in a 25 µL reaction, containing 12.5 µL of 10X PCR Buffer, 1 µL of 50 mM MgCl_2_, 0.5 µL of dNTPs (Fisher Scientific, Leichestershire, UK), 0.3 µL of each primer (20 µM), 0.2 µL of Platinum Taq DNA polymerase (Thermo Fisher Scientific, Waltham, MA, USA), and 3 µL of 50 ng of the template DNA and DNAse/RNAse free water up to the volume. The thermal profile included an initial denaturation at 94 °C for 2 min, followed by 32 cycles of denaturation at 94 °C for 1 min, annealing at 56 °C for 1 min, extension at 72 °C for 1 min, and final extension at 72 °C for 10 min. Amplicons were run on 2% agarose gel and visualized under UV exposure by a transilluminator. A 50–2000 kb ladder was used as a molecular marker (Bio-Rad, Hercules, CA, USA).

PCR products were purified with an ExtractMe DNA Clean-up and Gel-out kit (Blirt, Poland) according to the manufacturer’s instructions. The purified PCR products were bidirectionally sequenced using the same primer sequences used for the PCR, the Brilliant Dye Terminator (v1.1) Cycle Sequencing Kit (Thermo Fisher Scientific, Waltham, MA, USA) and Dye Ex 2.0 spin kit (Qiagen, Venlo, The Netherlands) in Seqstudio genetic analyzer (Applied biosystems, Woburn, MA, USA). DNA sequence analyses were performed in DNASTAR Lasergene Software. Retrieved amplicon sequences were compared to the GenBank database using the Basic Local Alignment search Tool (BLAST) search algorithm.

### 2.6. Serotype

Serotype was investigated using the primers LGD-F (5′-GGATGGAACTTCCTGCCACA-3′) and LGD-R (5′-ATCCTTGAGAACGAAGG-3′) as described by Ohbayashi et al. [36]. Amplification was performed in a final volume of 25 µL, containing 12.5 µL of Platinum^TM^ PCR Supermix-UDG (Thermo Fisher Scientific), 0.5 µL of each primer (10 µM), 5 µL of 50 ng of the template DNA and RNase and DNase free water up to the volume.

The amplification conditions included a first step at 50 °C for 2 min, a denaturation at 95 °C for 2 min, followed by 32 cycles of denaturation at 95 °C for 30 s, annealing at 55 °C for 30 s, extension at 72 °C for 1.30 min, and final extension at 72 °C for 7 min. Amplicons were analyzed on a 2% agarose gel and visualized under UV exposure by a transilluminator. A 50–2000 kb ladder was used as a molecular marker (Bio-Rad). Serotype I was characterized by a 285 bp, while serotype II by a 1285 bp band.

### 2.7. Detection of Virulence Factors

The following virulence genes was investigated through two different multiplex PCR and one simplex PCR: *NADH oxidase*, *LPxTG3*, *adhPsaA*, *pgm*, *adhPav*, *LPxTG2*, *Adhesion*, *AdhCI*, *AdhCII*, *sod*, *eno*, *Hly1* as previously described by Salighehzadeh et al. [13].

The first multiplex PCR targeting *NADH oxidase*, *LPxTG3*, *adhPsaA*, *pgm* genes was performed in a final volume of 40 µL, containing 20 µL of UCP-Hi Master Mix (Qiagen), and primers used at a final concentration of 0.25 mM including; 0.5 µL of *NADH oxidase* primer (F and R), 0.7 µL of *LPxTG3* primer (F and R), 0.7 µL of *adhPsaA* primer (F and R), 0.9 µL of *pgm* primer (F and R), 5 µL of 50 ng of the template DNA and RNase and DNase free water up to the volume. The thermal profile included an initial denaturation at 98 °C for 30 s, followed by 34 cycles of denaturation at 94 °C for 50 s, annealing at 56.8 °C for 1 min, extension at 72 °C for 1 min, and final extension at 72 °C for 7 min. The second multiplex PCR targeting *AdhCI*, *AdhCII*, *sod*, *eno* genes was performed in a final volume of 25 µL, containing 12.5 µL of Mastermix, 0.5 µL of each primer (10 µM), 5 µL of 50 ng of the template DNA and RNase and DNase free water up to the volume. The thermal profile included an initial denaturation at 95 °C for 2 min, followed by 34 cycles of denaturation at 94 °C for 50 s, annealing at 56.8 °C for 1 min, extension at 72 °C for 1 min, and final extension at 72 °C for 7 min.

The simplex PCRs targeting *adhPav*, *LPxTG2*, *Adhesion* genes were performed in a final volume of 25 µL, containing 12.5 µL of Mastermix, 0.5 µL of each primer (10 µM), 5 µL of 50 ng of the template DNA and RNase and DNase free water up to the volume. The thermal profile included a first step at 50 °C for 2 min, an initial denaturation at 95 °C for 2 min, followed by 34 cycles of denaturation at 94 °C for 50 s, annealing at 56.8 °C for 1 min, extension at 72 °C for 1 min, and final extension at 72 °C for 7 min. For all PCR reactions, amplicons were analyzed on a 2% agarose gel for quantification and visualized under UV exposure by a transilluminator. A 50–2000 kb ladder was used as a molecular marker (Bio-Rad, Hercules, CA, USA).

In addition to the virulence genes listed above, amplification of capsule cluster genes and the three hemolysin genes (*Hly1*, *Hly2* and *Hly3*) were amplified following the protocol previously described [37,38]. Amplicons were run on a Gelgreen (Biotium, Fremont, CA, USA) stained agarose gel and then visualized under UV exposure. A 50–2000 kb ladder (Amplisize Molecular Ruler, Bio-Rad, Hercules, CA, USA) was used as a molecular marker. For comparison, the *Lactococcus petauri* isolate 33932 1LP_H was used in the PCR studies.

### 2.8. Phylogeny

The evolutionary history of the sequenced amplicons was inferred using the Neighbor-Joining method [39]. The percentage of replicate trees in which the associated taxa clustered together was determined performing a bootstrap test (1000 replicates) [40]. The evolutionary distances were computed using the Maximum Composite Likelihood method [41]. This analysis involved 25 nucleotide sequences in total (13 publicly available DNA sequences of *Lactococcus petauri* deriving from GenBank collection and 2 obtained in the present study; 10 publicly available DNA sequences of *Lactococcus garvieae* deriving from GenBank collection; *Lactococcus lactis* DNA sequence was instead used as outgroup). All ambiguous positions were removed for each sequence pair (pairwise deletion option). There was a total of 535 positions in the final dataset. Evolutionary analyses were conducted in MEGA X [42].

## 3. Results

### 3.1. Thematic Networks of Lactococcus spp. Keywords Revealed by VOSviewer

**Figure 1:**
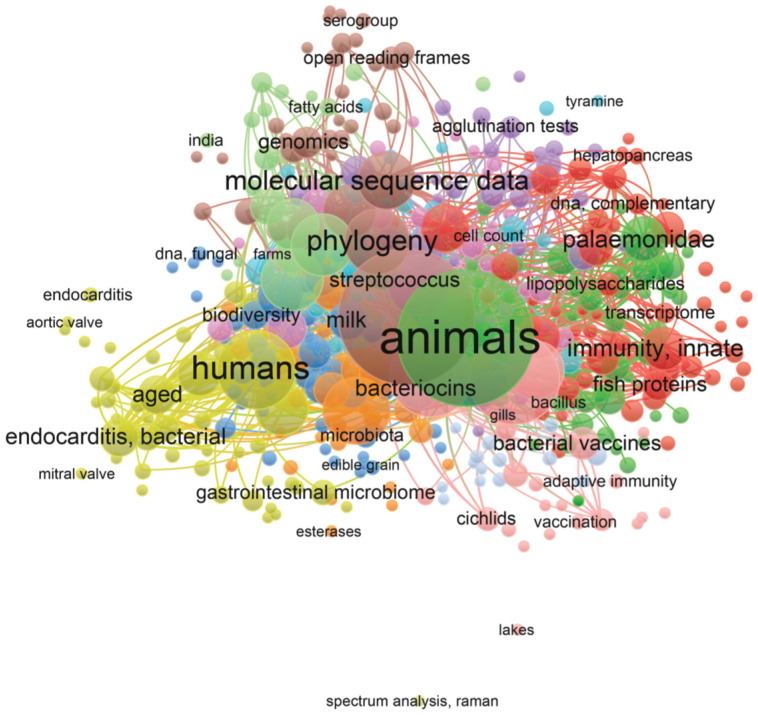
Visualization of bibliometric analysis on *Lactococcus garvieae* literature. Figure illustrates keyword co-occurrence networks, highlighting associations among various keywords used in the analyzed documents. The analysis provides insights into thematic connections and emerging trends within the scientific literature. Modified graphs were generated using VOSviewer software (version 1.6.20) [30].

**Figure 2:**
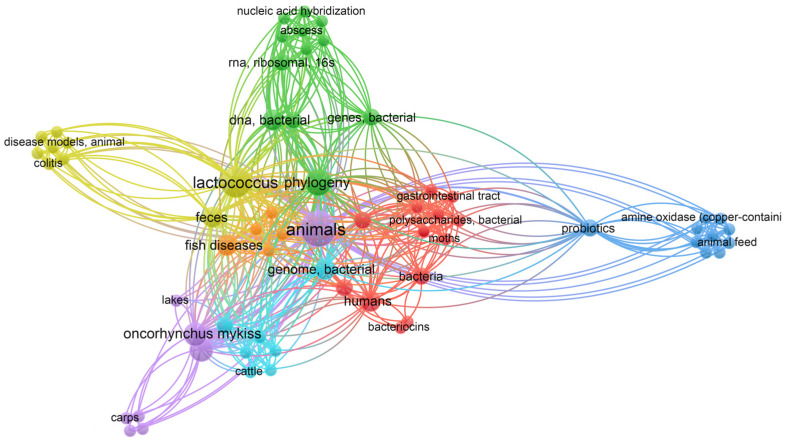
Visualization of bibliometric analysis on *Lactococcus petauri* literature. Figure illustrates keyword co-occurrence networks, highlighting associations among various keywords used in the analyzed documents. The analysis provides insights into thematic connections and emerging trends within the scientific literature. Modified graphs were generated using VOSviewer software (version 1.6.20) [30].

Bibliometric analysis conducted using VOSviewer allowed us to visualize a co-occurrence network of keywords related to *Lactococcus garvieae* and *Lactococcus petauri* (Figure 1 and Figure 2, respectively; Supplementary Figures S1 and S2). The nodes in the map represent different keywords used, while the lines connecting the nodes indicate how frequently these keywords appear together in the analyzed documents. Node size and line density highlight the importance and close relationships between the keywords.

Several distinct clusters emerged from the analysis, each representing a specific sub-theme within research on *Lactococcus* spp. For instance, the prominent “Animals” cluster highlights research on infections in aquatic species such as trout, as well as infections in other terrestrial animals, while another large cluster, “Human”, focuses on infections in humans (Figure 1).

The results indicate a significant increase in scientific interest towards these two species belonging to the genus *Lactococcus* in recent years (Supplementary Figures S1 and S2), with growing attention to their relevance not only in aquaculture but also in veterinary and human medicine. This trend reflects the importance of monitoring and managing infections caused by this species, given its ability to affect a wide range of hosts [7,12,14,28].

### 3.2. Microbial Identification

The predominant growing colonies from both cases were identified as round, smooth, non-pigmented, and non-hemolytic colonies after 2–3 days of incubation. Using MALDI-TOF MS on selected colonies form both cases identified the bacteria as *Lactococcus garvieae*. The bacterial isolates were assigned as LP1D and LP2-D from case 1 and 2, respectively. Some colonies from case 2 were also identified as *Citrobacter braaki* and *Enterobacter ludwigi*.

### 3.3. Antibiotic Susceptibility

Table 1 summarize antimicrobial patterns of the two canine strains. Recommended antimicrobials for *Lactococcus garvieae* are ampicillin-AM, amoxicillin-AX, ceftriaxone-CTX (or vancomycin-VAN in monotherapy or in combination with gentamicin-GEN) while *Lactococcus petauri* is not mentioned. Significant resistance was reported only for clindamycin-DA.

### 3.4. PCR and Sequencing of ITS 16S-23S Region

BLAST analysis for urine sample from case 1 (ID #49222) and skin tissue sample from case 2 (ID #50963) revealed an identity percentage of 100% with *Lactococcus petauri* sequences isolated from rainbow trout in Spain, Turkey and Greece, deposited in GenBank database (Accession numbers: OQ108346, OQ108345 and OQ108344, respectively). Amplicon sequences retrieved from this study were deposited in GenBank public database as 1LP_D (ID #49222) and 2 LP_D (ID #50963) for the isolate from skin lesion and GenBank accession numbers were assigned as PQ142844 and PQ142845, respectively.

### 3.5. Serotype PCR

**Figure 3:**
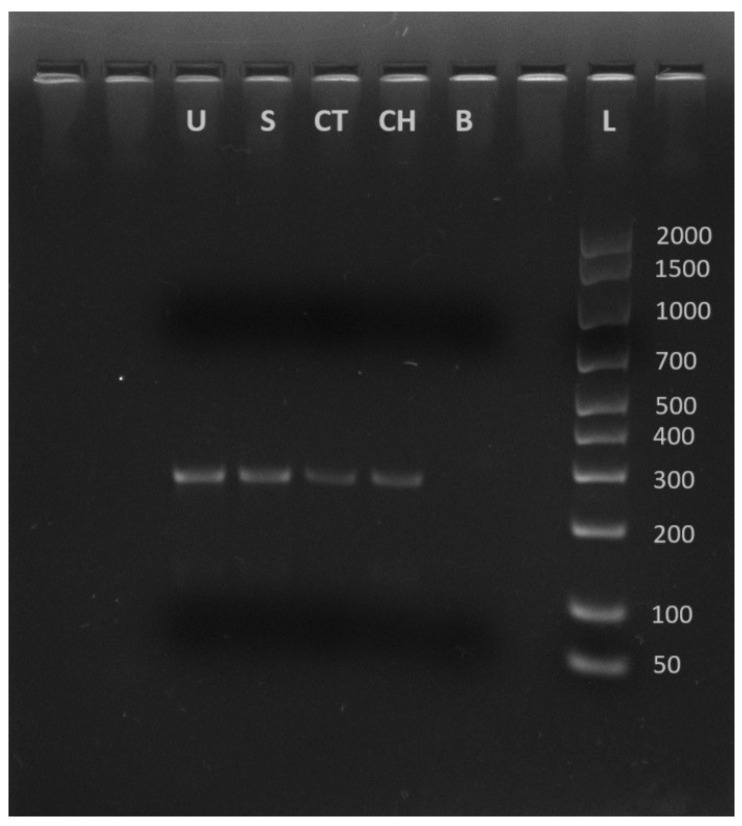
U = urine sample; S = skin sample; CT = positive control (positive field control from trout *Lactococcus petauri*); CH = positive control (human positive for *L. petauri* 1LP_H GenBank Accession number OR231105); B = non template control (ddH2O only); L = ladder (50–2000 bp).

Both samples showed a band of 285 bp confirming the presence of serotype I (Figure 3). This results also matched the serotype result obtained for the *Lactococcus petauri* isolate previously recovered from human urinary tract infection [28].

### 3.6. Virulence Factors

All the samples were positive for *AdhCI*, *sod*, *adhPsaA*, *Hly2*, *adhPav* genes and negative for *AdhCII*, *Hly1* and capsule gene cluster *CGC*. The same factors have been reported also for the *Lactococcus petauri* strain 33932 1LP_H. Few factors were found positive only for the human isolate such as: *eno*, *NADH oxidase*, *pgm* and *Adhesin*. On the other hand, only *LPxTG3* was found positive for canine isolates and negative for the human one. *Hly3* and *LPxTG2* were positive only for canine isolate 49222 1LP_D. Results of the virulence factor investigation in *L. petauri* isolates recovered in this study are summarized in Table 2.

### 3.7. Phylogeny

The phylogenetic tree showed three main clusters subdivided by species; one for *Lactococcus garvieae*, one for *Lactococcus petauri* containing also the two sequences isolated from dogs and the human isolate from urinary infection and a final one was made of only *L. lactis* inserted as outgroup.

**Figure 4:**
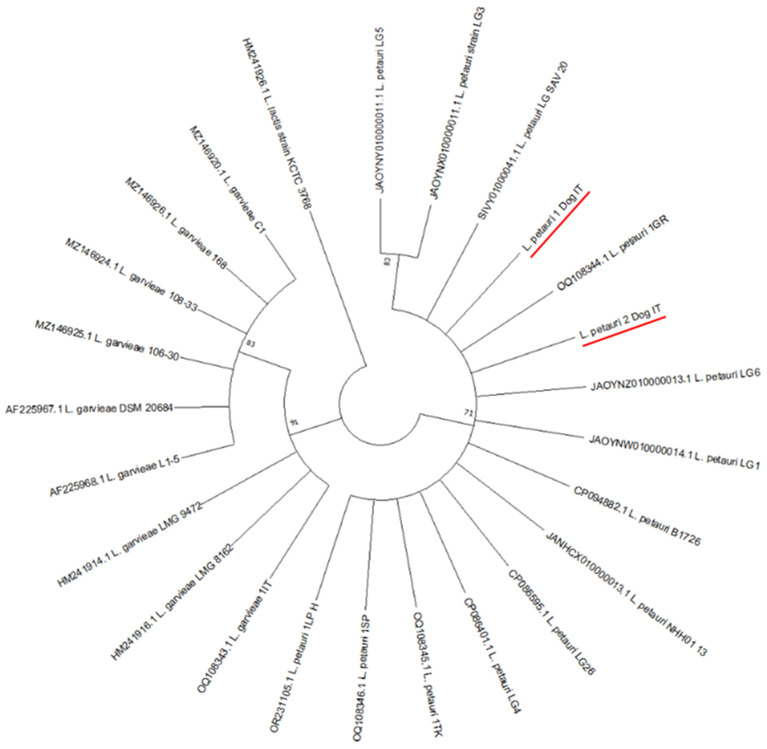
Phylogenetic tree based on neighbor-joining method with bootstrap support values, the underlined sequences represent our data.

The optimal tree obtained using the neighbor-joining method is shown in Figure 4. The percentage of replicate trees in which the associated taxa clustered together in the bootstrap test (1000 replicates) are shown next to the branches.

## 4. Discussion

The number of scientific studies on *Lactococcus garvieae*, initially identified as a human pathogen, has seen a significant expansion since 1991. Early research focused on the identification and characterization of *L. garvieae* in human contexts, with the first study by Elliot et al. [43] differentiating *L. lactis* from *L. garvieae* based on whole-cell protein patterns. During the initial years, the number of studies was limited, with only a few papers published annually until the mid-1990s.

In 1996, a major shift occurred with the beginning of research on *L. garvieae* in aquatic organisms, particularly in freshwater fish like rainbow trout (*Oncorhynchus mykiss*), as highlighted by Eldar et al. [44] in Italy. During this period, pathogen identification techniques improved, leading to increased studies on marine species such as amberjack (*Seriola* spp.) and crustaceans like giant freshwater prawn (*Macrobrachium rosenbergii*), with the first experimental studies conducted in 1999 [45,46].

The total number of studies steadily grew, reaching 125 publications between 2000 and 2010, with an average of 13 studies per year. Major research topics during this decade included infections in aquatic organisms and septicemia in immunosuppressed humans [47], along with the characterization of the pathogen in dairy and meat products [48]. A significant peak was observed in 2006, with 22 studies, the highest in that decade, covering topics such as human endocarditis and the biodiversity of *L. garvieae* in Mediterranean fish [49].

After 2010, the trend showed further growth, with a total of 269 studies on *L. garvieae* related to aquatic and human organisms, accounting for roughly 54.0% of the overall literature. In the following decade, the number of publications continued to increase, culminating in a peak of 45 studies in 2023. This exponential growth can be attributed to the diversification of studied species and topics, including fish, crustaceans, mammals, and even dogs, with the first study on *L. garvieae* isolated from canine tonsils published in 1996 [11]. The research expansion also extended to aquaculture and human health sectors, with investigations into food products and infectious diseases, as shown by studies published between 2011 and 2021.

In conclusion, the field of research on *L. garvieae* has undergone significant evolution, from being identified as a human pathogen to becoming an important aquatic and animal pathogen, with a continuous increase in studies and a clear thematic diversification.

In this context, the discovery of *Lactococcus petauri* in 2017, first isolated from a sugar glider (*Petaurus breviceps*) abscess [5], marked an important milestone. After a two-year gap with no published research, studies resumed in 2020, focusing on genomic analyses in both human gut microbiota and rainbow trout lactococcosis outbreaks [23,50]. These early studies set the stage for further investigations into the role of the bacterium in both human health and aquaculture.

In 2021, research remained steady with topics such as its probiotic potential and occurrence in traditional cheese [51,52]. However, 2022 marked a turning point, with a 100% increase in studies. New research included the reclassification of strains, the presence of the bacterium in feed insects, and insights into bacteriocin production and fish immune responses [21,53,54].

By 2023, research efforts reached a new peak with five studies, diversifying further into new topics, including environmental monitoring of *L. petauri* in Californian lakes [29], human urinary tract infections [28], and vaccine efficacy in rainbow trout [55]. The virulence of the pathogen at varying temperatures and species susceptibility were also investigated [56], and new molecular diagnostic tools were developed to differentiate between *L. garvieae* and *L. petauri* [22]. This period highlights the emerging significance of the pathogen in both human health and aquaculture, with a particular focus on diagnostic advancements.

Subsequently, in 2024, research continued with studies on bacteriocin production, effects on inflammation and gut health, infective endocarditis, and genomic comparisons with other *Lactococcus* species [7,57,58,59,60]. The trend reveals a growing focus on the significance of *L. petauri* in aquaculture, accounting for 42.1% of studies, particularly in rainbow trout (31.6%). While human-related research also increased (21.0%), no studies have yet explored terrestrial animals, such as domestic dogs.

Advances in molecular techniques have deepened our understanding of this emerging pathogen [26], and climate change is expected to further drive its spread and shape future research. In this context, the present study represents the first report of *L. petauri* infection in dogs. The application of a new molecular diagnostic tool, specifically sequencing of the ITS region, enabled the correct identification of two strains that had previously been misidentified as *L. garvieae* using routine methods such as MALDI-TOF MS. Additionally, phylogenetic analysis confirmed that these two strains cluster within the *L. petauri* group, which is clearly distinct from *L. garvieae*.

The route of infection in these cases is unknow. *L. petauri* is a member of the intestinal microbiota of healthy human gut and none of the cases of *L. petauri* bacteremia described by Chan et al. [7] were associated with seafood consumption before the appearance of the symptoms. This suggests that *L. petauri* infections in humans could arise from pre-existing gut colonization rather than recent exposure to contaminated food sources and the same hypothesis could be valid for two cases reported in dogs.

Difference in virulence gene composition was observed according to the species and the source [61]. For that purpose, we tried to compare not only the two canine isolates but also extend the comparison to include a human isolate *of L. petauri.*

The canine *L. petauri* strains, the human one and *L. garvieae* Bacchus described by Thiry et al. [12] were all not capsulated confirming that capsule genes cluster (CGC) might not be responsible for the virulence even if it contributes to the increasing of bacterial resistance to phagocytosis and other factors should be considered [37].

Adhesins are crucial for bacterial colonization, as they facilitate attachment to the host cell surface and help evade clearance by mucosal secretions and peristalsis. Different types of adhesins may be expressed to achieve this. *Adhesin cluster I, II, Adhesin Psa A, Adhesin Pav* and *Adhesin* were analyzed and *Adhesin cluster I, Psa A, Pav* were all positive in human and canine *L. petauri*. Putative adhesine analyzed by Thiry et al. [12] was positive also for *L. garvieae* Bacchus, confirming the importance of these virulence factor independently from the bacterial species.

Superoxide dismutases (SODs) are enzymes with a strong anti-inflammatory power and could be synthetized by bacteria to protect themselves. All the strains screened in our study were positive for *Sod* including Bacchus.

LpxTg are surface proteins bind to the peptidoglycan layer and play an important role in bacterial virulence. In this study, only *LpxTg2* and *3* have been investigated. They have been detected in canine isolates *of L. petauri* where *LpxTg3* was found in both isolates while *LpxTg2* was detected only in isolate 49222. This result is supported by the study of Thiry et al. [12] where *LpxTg2* and *3* were also detected for canine *L. garvieae*.

The role of Enolase, Phosphoglucomutase and NADH oxidase is not known yet. Enolase is a cytoplasmic and a surface-associated protein in bacteria, it is involved in plasmin activation on the bacterial surface favoring bacterial migration in the host by damaging tissue barriers [62]. Phosphoglucomutase in bacteria is known to play roles in biosynthesis of multiple exoproducts, including lipopolysaccharide and lipoteichoic acid and is involved in the maintenance of the cell wall properties and antibiotic resistance [63]. NADH oxidase has been reported to interact with fibronectin of the host cells and induce cellular oxidative stress and apoptosis [64].

In our study, *Enolase*, *Phosphoglucomutase* and *NADH oxidase* were positive only for *L. petauri* human isolate but negative for the investigated canine isolates. This may indicate differences in virulence between these isolates.

Hemolysins cause the lysis of red and white blood cells [37]. In our study, the presence of three hemolysin genes (*hly1*, *hly2* and *hly3*) was analyzed, however, only *hly2* was found positive in all the isolates while *hly1* on the contrary was negative. *Hly3* was positive only for 49222 canine isolate and for *L. garvieae* isolate Bacchus.

Concerning the serotypes, all the isolates were found to belong to serotype I which is the most common among *L. garvieae* and *L. petauri*. Serotype II has recently been attributed by Mahmoud et al. [4] to the species *Lactococcus formosensis*.

Comparing the two canine isolates, *hemolysin 3* and *Lpx Tg2* were amplified only for 49222, suggesting the higher virulence of this strain. This seems to be in accordance with the isolation of *L. petauri* (49222) as a unique agent in urine, which had similar clinical signs to those observed; other colonies, identified as *Citrobacter braaki* and *Enterobacter ludwigi,* were instead isolated with *L. petauri* (50963) from skin interdigital nodules, preventing us from understanding the real contribution of each bacterium to the clinical signs observed. Chan et al. [7] screened 120 isolates of *L. petauri* from various origins including, 13 from human infections, 58 from human feces, 35 from rainbow trout, 4 from bovines, and 10 from other unspecified sources for virulence factors. The following percentages of virulence factors were reported: *AdhCI* 98%, *Sod* 100%, *AdhCII* 88%, *eno* 96%, *Hly1* 100%, *NADH oxidase* 100%, *Hly3* 100%, *LpxTg3* 17%, *Adh PsaA* 100%, *Hly 2* 100%, *pgm* 100%, *Adh Pav* 100%, *LpxTg2* 44%, *Adh* 0%, *CGC* 5%. Our results align with previous reports in some cases, specifically regarding *sod*, adh1, *Adh PsaA*, and *Hly 2*. However, only 10 of the strains were from different origins and likely not sourced from dogs. Given the limited number of strains available in this study, it was not possible to unequivocally associate specific virulence factors with a particular species or host. However, certain factors, such as adhesins and SOD, appear to be more crucial than others.

The antibiotic results reveal significant differences between animal and human strains, particularly regarding sensitivity to tetracycline and sulphonamides. Specifically, the human samples demonstrated clear resistance to tetracycline (MIC 256 μg/mL, human strain), while the canine strains were sensitive (MIC 1 μg/mL). This suggests a potential difference in resistance mechanisms between the two species, possibly linked to the more frequent use of these antibiotics in humans.

Regarding folate pathway inhibitors, the human strain showed resistance to trimethoprim/sulfadiazine (MIC > 32 μg/mL), while the canine samples were susceptible to this antibiotic. This discrepancy may reflect the limited use of this class of antibiotics in pets’ veterinary treatments compared to food animals and human medicine, influencing the development of specific resistances. Thus, more surveys are warranted to characterize *L. petauri* isolates from canine origins in comparison to similar species from other mammalian and non-mammalian origins.

## 5. Conclusions

This paper presents the first documented case of *Lactococcus petauri* infection in domestic dogs, where the bacterium was identified in urine and skin samples.

The growing interest in *Lactococcus garvieae* and *L. petauri* is evident from the literature, driven by their significant impact not only in aquaculture but also in other fields such as veterinary and human medicine. This trend underscores the importance of monitoring and managing infections caused by these species due to their broad host range and the emerging role of *L. garvieae* as a zoonotic agent. This study provides new insights into the host tropism of these long-misclassified species and contributes valuable data on their virulence factors and antimicrobial resistance. To further elucidate how virulence factors may be adapted to canine hosts and to track antimicrobial resistance trends in *Lactococcus* isolates from pets, it is essential to examine more isolates from canine origins.

## Figures and Tables

**Table 1:** Pattern of antibiotic susceptibility of *Lactococcus petauri* isolated from canine skin and urine samples, compared with a human strain isolated from urine by Colussi et al. [28].

Antibiotic.		Urine			Skin Swab	
	49222 Dog 1 (This Study)		33932 Human [28]		50963 Dog 2 (This Study)	
	MIC Value (μg/mL)	Sample Sensitivity	MIC Value (μg/mL)	Sample Sensitivity	MIC Value (μg/mL)	Sample Sensitivity
Amoxicillin/Clavulanic acid	1	NI	−	−	2	NI
Ampicillin	1	S	0.5	S	1	S
Cefalexin	>8	NI	−	−	−	−
Cefazolin	4	NI	−	−	4	NI
Ceftiofur	−	−	−	−	1	NI
Cefpodoxime	≤2	NI	−	−	−	−
Ceftriaxone	−	−	0.25	S	−	−
Doxycycline	0.5	NI	−	−	−	−
Enrofloxacin	1	NI	−	−	1	NI
Erythromycin	≤0.12	S	−	−	0.06	S
Florfenicol	≤4	NI	−	−	−	−
Gentamicin	4	NI	−	−	−	−
Kanamycin	>16	NI	−	−	≤8	NI
Kanamycin High Level	−	−	−	−	≤250	NI
Levofloxacin	−	−	1	S	−	−
Meropenem	−	−	0.032	S	−	−
Oxacillin + 2% NaCl	>8	NI	−	−	>4	NI
Penicillin	1	S	0.5	S	2	I
Rifampin	−	−	−	−	>2	NI
Sulfisoxazole	−	−	−	−	>512	NI
Tetracycline	1	S	256	R	1	S
Tilmicosin	−	−	−	−	16	NI
Trimethoprim/Sulphadiazine	0.5	S	>32	R	0.5	S

Interpretative criteria of CLS M45 3rd edition [34]. R = resistant; I = intermediate; S = sensitive; NI = not interpretable.

**Table 2:** Virulence genes amplification.

Target Gene	Sample ID		
	33932 1LP_H	49222 1LP_D	50963 2LP_D
*Adhesin Cluster 1 (AdhCI)*	+	+	+
*Superoxide dismutase (sod)*	+	+	+
*Adhesin Cluster 2 (AdhCII)*	*-*	-	-
*Enolase (eno)*	*+*	-	-
*Hemolysin 1 (Hly1)*	*-*	-	-
*NADH oxidase*	*+*	-	-
*Hemolysin 3 (Hly3)*	*-*	+	-
*Lpx Tg3*	*-*	+	+
*Adhesin Psa A (adhPsaA)*	*+*	+	+
*Hemolysin 2 (Hly2)*	*+*	+	+
*Phosphoglucomutase (pgm)*	*+*	-	-
*Adhesin Pav (adhpav)*	*+*	+	+
*Lpx Tg2*	*-*	+	-
*Adhesin*	*+*	-	-
*Capsule gene cluster (CGC)*	*-*	-	-

+ = presence of the target gene in the sample; - = absence of the target gene in the sample.

## Data Availability

ITS sequencing of the two *Lactococcus petauri* canine isolates have been deposited to GenBank (https://www.ncbi.nlm.nih.gov/genbank/, accessed on 1 September 2024) with the following accession numbers: PQ1428441 and PQ142845, respectively, for LP_D (49222) and 2 LP_D (50963).

## Supplementary Materials

The following supporting information can be downloaded at: https://www.mdpi.com/article/10.3390/ani14223279/s1, Figure S1: Temporal trend of bibliometric analysis on *Lactococcus garvieae* literature; Figure S2: Temporal trend of bibliometric analysis on *Lactococcus petauri* literature.
