# Supplementary material for: First Detection of Lactococcus petauri in Domestic Dogs in Italy
Source: Animals (Basel). 2024 Nov 14;14(22):3279. doi: 10.3390/ani14223279 (PMC11591435; doi:10.3390/ani14223279)
Supplement: Supplementary file 1 [file animals-14-03279-s001.zip › animals-3235742-supplementary.pdf]

SUPPLEMENTARY MATERIALS

# First Detection of *Lactococcus petauri* in Domestic Dogs in Italy

**Simona Sciuto [1], Giuseppe Esposito [1,*], Paolo Pastorino [1], Khalid Shahin [2], Katia Varello [1], Eliana Trabunella [1], Giulia Milanese [1], Sonia Scala [1], Marino Prearo [1], Pier Luigi Acutis [1], Angelo Salerno [3], Simona Zoppi [1] and Silvia Colussi [1,*]**

[1] Istituto Zooprofilattico Sperimentale del Piemonte, Liguria e Valle d'Aosta, 10154 Turin, Italy; simona.sciuto@izsplv.it (S.S.); paolo.pastorino@izsplv.it (P.P.); katia.varello@izsplv.it (K.V.); eliana.trabunella@izsplv.it (E.T.); giulia.milanese@izsplv.it (G.M.); sonia.scala@izsplv.it (S.S.); marino.prearo@izsplv.it (M.P.); pierluigi.acutis@izsplv.it (P.L.A.); simona.zoppi@izsplv.it (S.Z.)
[2] PHARMAQ Analytiq UK Ltd., 8b Solasta House, Inverness Campus, Scotland IV2 5NA, UK; khalid.shahin@zoetis.com
[3] S.S. Microbiologia, Ospedale SS Antonio e Margherita, 15057 Tortona, Italy; asalerno@aslal.it
* Correspondence: giuseppe.esposito@izsplv.it (G.E.); silvia.colussi@izsplv.it (S.C.)

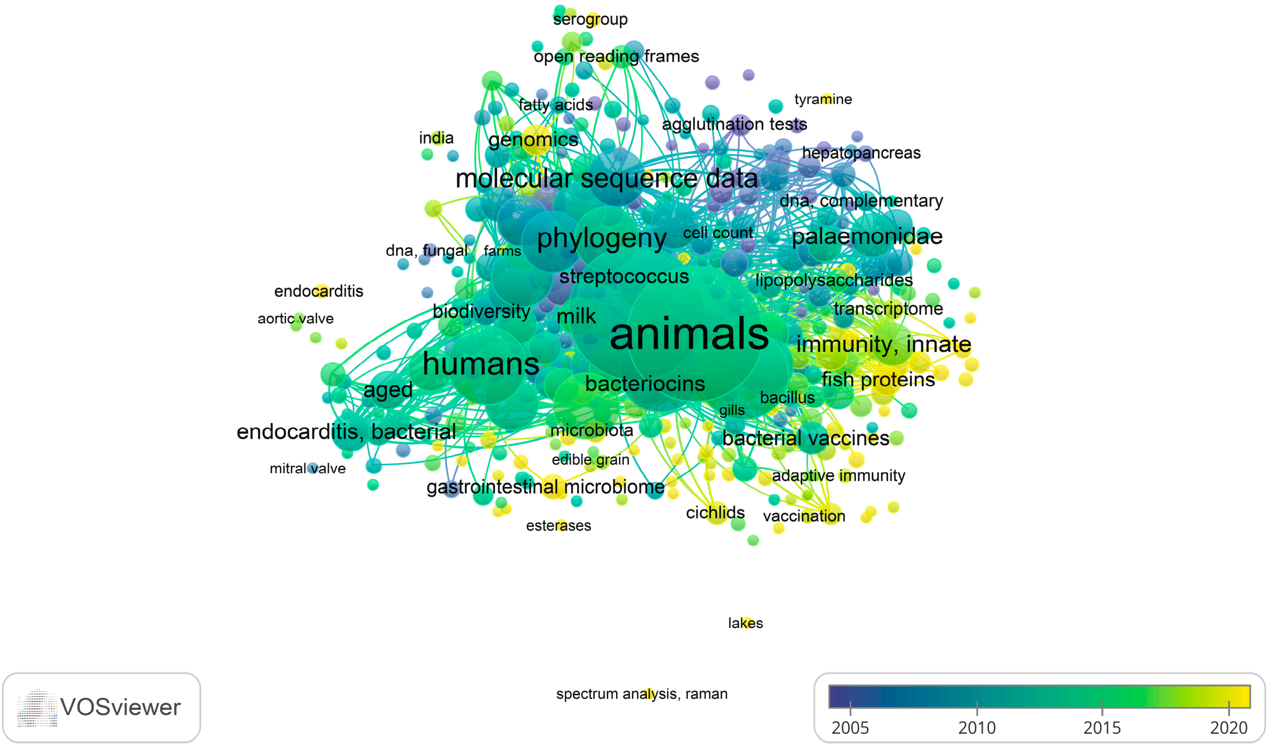


**Supplementary Figure S1**. Visualization of bibliometric analysis on *Lactococcus garvieae* literature. The figure illustrates keyword co-occurrence networks, highlighting associations among various keywords used in the analysed documents. The analysis provides insights into thematic connections and emerging trends within the scientific literature. Graphs were generated using VOSviewer software (version 1.6.20) [1].

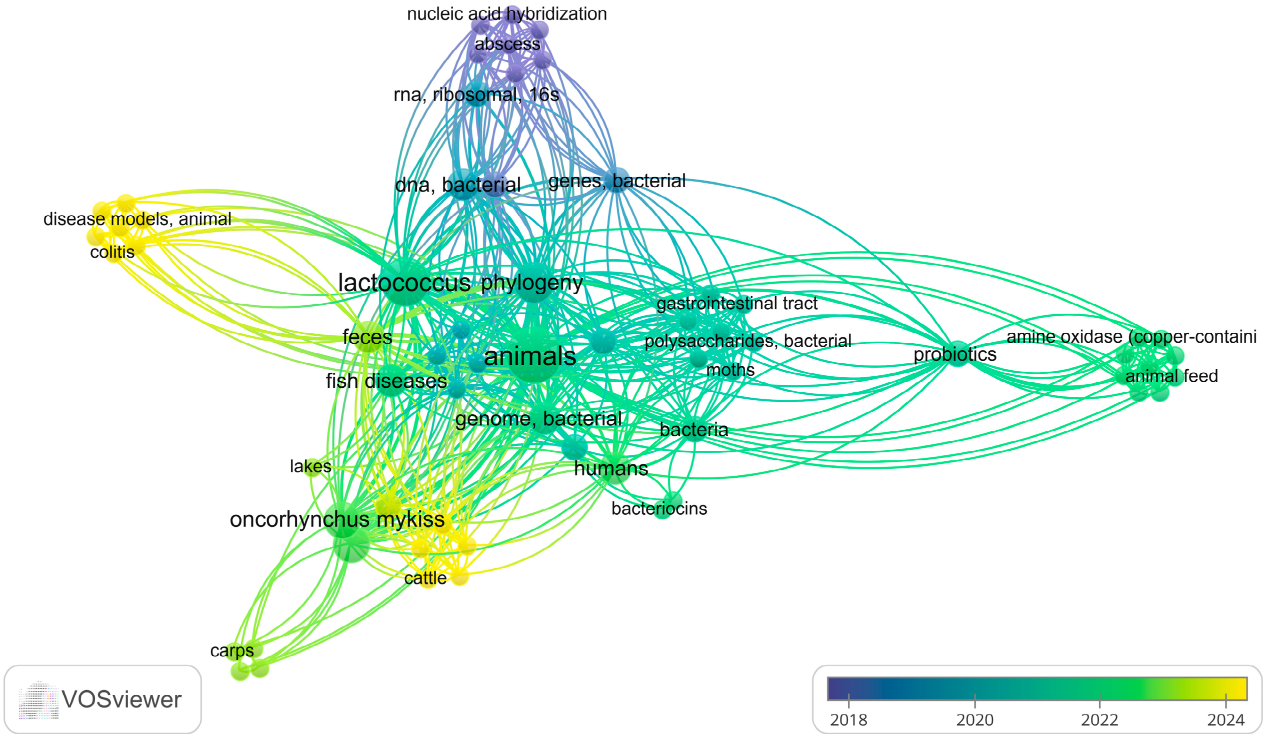


**Supplementary Figure S2**. Visualization of bibliometric analysis on *Lactococcus petauri* literature. The figure illustrates keyword co-occurrence networks, highlighting associations among various keywords used in the analysed documents. The analysis provides insights into thematic connections and emerging trends within the scientific literature. Graphs were generated using VOSviewer software (version 1.6.20) [1].

## Reference

1. Van Eck, N.; Waltman, L. Software survey: VOSviewer, a computer program for bibliometric mapping. *Scientometrics*, **2010**, *84(2)*, 523-538. https://doi.org/10.1007/s11192-009-0146-3.
